# Causal association between major depressive disorder and coronary heart disease: a two-sample bidirectional mendelian randomization study

**DOI:** 10.1186/s12920-023-01625-5

**Published:** 2023-08-08

**Authors:** Qianjie Xu, Chen Chen, Ruijia You, Linghao Ni, Siyu Chen, Bin Peng

**Affiliations:** Department of Health Statistics, School of Public Health, Chongqing Medical University, Chongqing, 400016 China

**Keywords:** Major depressive disorder, Coronary heart disease, Causal inference, Bidirectional causality, Mendelian Randomization

## Abstract

**Background:**

Major depressive disorder (MDD) is a highly heterogeneous mental illness and a major public health problem worldwide. A large number of observational studies have demonstrated a clear association between MDD and coronary heart disease (CHD), and some studies have even suggested that the relationship is bidirectional. However, it was unknown whether any causal relationship existed between them and whether causality was bidirectional in such an instance. Thus, we aimed to determine whether there is a bidirectional causal relationship between major depressive disorders and coronary heart disease.

**Methods:**

Our two-sample Bidirectional Mendelian Randomization Study consisted of two parts: forward MR analysis regarded MDD as exposure and CHD as the outcome, and reverse MR analysis considered CHD as exposure and MDD as the outcome. Summary data on MDD and CHD were obtained from the IEU Open GWAS database. After screening criteria(*P* < $5\times {10}^{-8}$), 47 MDD-associated SNPs and 39 CHD-associated SNPs were identified. The inverse-variance weighted (IVW) method, ME-Egger regression, and weighted median method were used to estimate causality. In addition, sensitivity methods, including the heterogeneity test, horizontal pleiotropy test, and leave-one-out method, were applied to ensure the robustness of causal estimation.

**Results:**

Based on the MR-Egger regression intercept test results, there did not appear to be any horizontal pleiotropy in this study (MDD: intercept = -0.0000376, *P* = 0.9996; CHD: intercept = -0.0002698, *P* = 0.920). Accordingly, IVW results suggested consistent estimates of causal effect values. The results showed that people with MDD increased the risk of CHD by 14.7% compared with those without MDD (OR = 1.147, 95%CI: 1.045–1.249, *P* = 0.009). But there was no direct evidence that CHD would increase the risk of MDD(OR = 1.008, 95%CI: 0.985–1.031, *P* = 0.490). The heterogeneity test and funnel plot showed no heterogeneity in 47 SNPs of MDD (Q = 42.28, ${I}^{2}$=0, *P* = 0.629), but there was heterogeneity in 39 SNPs of CHD (Q = 62.48, ${I}^{2}$=39.18%, *P* = 0.007). The leave-one-out method failed to identify instances where a single SNP was either biased toward or dependent on the causation.

**Conclusion:**

Our study supports a one-way causal relationship between MDD and CHD, but there is no bidirectional causal relationship. MDD increases the risk of CHD, but there is no evidence that CHD increases the risk of MDD. Therefore, the influence of psychological factors should also be considered in the prevention and treatment of CHD. For MDD patients, it is necessary to prevent cardiovascular diseases.

## Introduction

Major depressive disorder (MDD) is a highly heterogeneous mental disorder characterized by long-term low mood, anhedonia, pessimism, and lack of initiative [1]. Studies demonstrate that MDD is a significant contributor to the global disease burden, affecting at least 350 million adults worldwide [2]. The prevalence of MDD is still rising yearly, making it a significant public health issue worldwide. Coronary heart disease (CHD) is an ischemic heart disease caused by atherosclerosis of the coronary arteries.

Specifically, a meta-analysis of the incidence of cardiovascular disease in comorbid MDD found that the prevalence of CHD in patients with MDD was 11.7% [2]. Another meta-analysis on CHD indicated that MDD was associated with a 1.5 to 2 times increased risk of CHD [3]. Additional research revealed that MDD was linked to a twofold risk of death after the diagnosis of coronary heart disease [4]. On the other hand, patients with CHD had an increased prevalence of depression as compared to the general population. It has been shown that MDD exists in 3.1% to 11.2% of patients with CHD in China [5]. According to an observational study, depression morbidity was discovered to be 10% in general community clinics, while rising to 30% in CHD clinics and as high as 50% in hospitalized patients undergoing coronary artery bypass surgery [3]. In addition, a further observational study revealed that antidepressant treatment in patients with CHD was related to a lower incidence of cardiovascular events, suggesting a possible bidirectional relationship between MDD and CVD [6]. Notably, the studies mentioned above only confirmed the correlation between MDD and CHD, discussing their risk factors instead of the causal relationship, particularly in regard to the potential bidirectional causal association between MDD and CHD.

Mendelian randomization (MR) research is a process of using single nucleotide polymorphisms (SNPs) as instrumental variables (IV) to establish models and infer and evaluate causal effects [7]. Due to the random distribution of alleles during the formation of human gametes, the MR method can overcome the limitations of confounding factors and reverse causality common in traditional observational studies. At the same time, genome-wide association study (GWAS) has developed rapidly in recent years, accumulating thousands or even millions of associations between genetic variations and phenotypes. Using these published data, the causal effects of risk factors on outcomes can be assessed without recruiting new patients or designing additional studies. Nowadays, MR research has been widely used in the field of causal inference.

This study aims to integrate multiple SNPs into IV by using MR, exploring the bidirectional causal relationship between MDD and CHD, as well as providing a scientific reference for the prevention and treatment of MDD and CHD.

## Materials and methods

### Study design

**Fig. 1:**
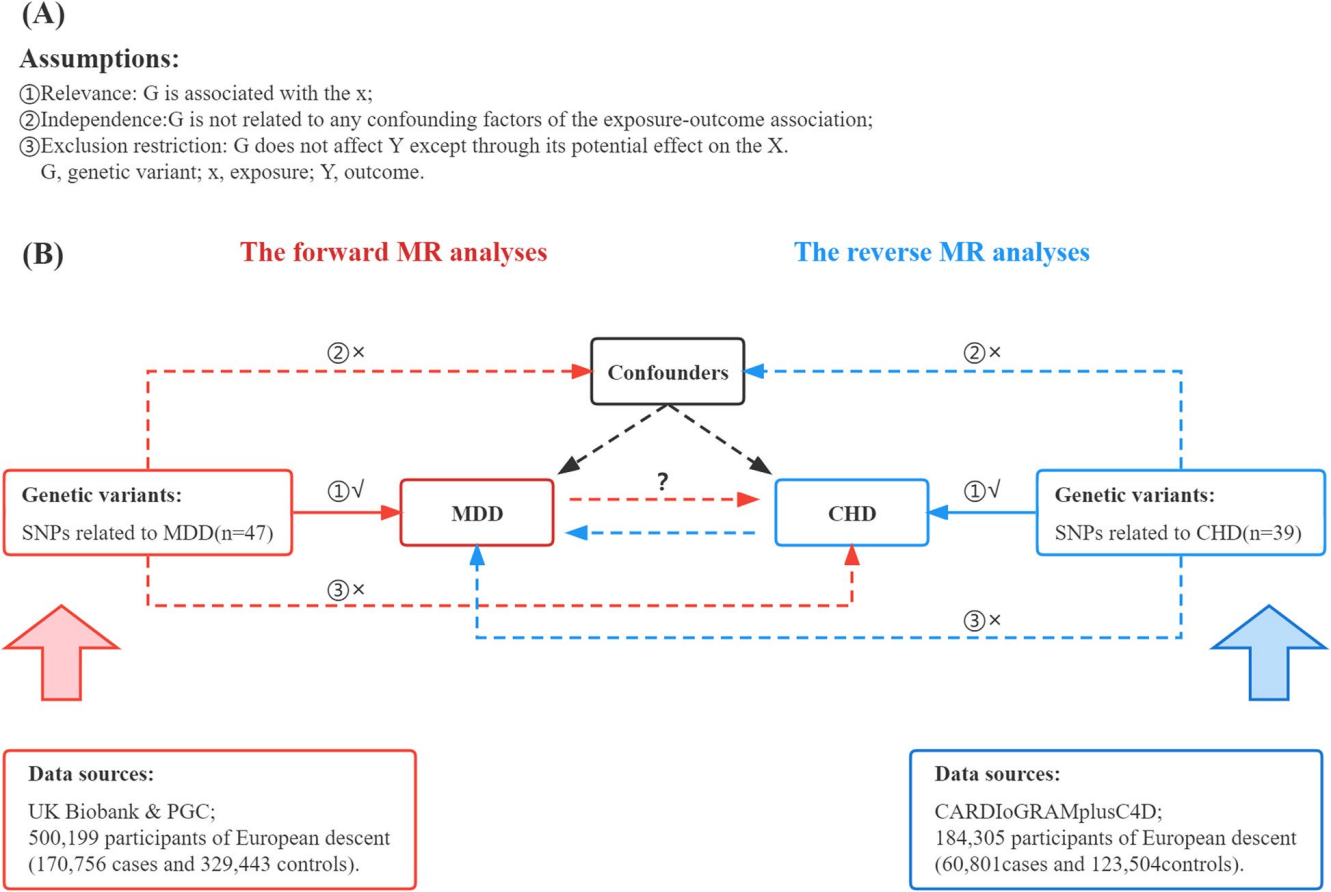
description of the study design in this bidirectional MR study. **A** MR analyses depend on three core assumptions. **B** Sketch of the study design. The red represented the forward MR analyses, with MDD as exposure and CHD as the outcome. The blue represented the reverse MR analyses, with CHD as exposure and MDD as the outcome. MDD, major depression disorder; CHD, coronary heart disease; MR, Mendelian randomization; SNPs, single‐nucleotide polymorphisms

Figure 1B shows a brief description of this bidirectional MR design between MDD and CHD. To investigate the bidirectional relationships between MDD and CHD, we performed two MR analyses by using summary data from GWAS. MR analysis of the forward side included exposure to MDD and CHD as an outcome, while the reverse analysis contained exposure to CHD and MDD as an outcome. Since our study was based on publicly available summary data, no ethical review was necessary.

When using MR for causal inference, genetic variation must satisfy three assumptions (Fig. 1A) in order to obtain a unbiased causal effect estimate [8]. For assumption one, we measured it by the F-statistic, F = $\frac{{R}^{2}(n-1-k)}{(1-{R}^{2})\times k}$, Where ${R}^{2}$ reflected the proportion of exposure variance that could be explained by genetic variation, which could be obtained by querying the MRBASE website (http://www.Mrbase.org/), n was the sample size, and k was the number of SNPs. It was generally considered that assumption one was satisfied when F > 10, which indicated no weak instrumental variable bias. Assumptions two and three were inherently untestable. Due to the random assignment of alleles to gametes, the second hypothesis of no association between IV and confounders was often considered to be satisfied, which was another advantage of the MR method [9]. The MR-Egger regression model's zero intercept term proved that hypothesis III was true.

### Data sources

The genetic variants associated with MDD came from a study of the genetic architecture of brain structure and function published in Nature in 2019 [10]. A total of 170,756 cases and 329,443 controls from the UK Biobank of European ancestry participated in the study. The number of SNPs in this study was 11,734,353. The genetic variation data of CHD were from a genome-wide association Meta-analysis of coronary artery disease published in Nature in 2015 [11]. This study included 60,801 cases and 123,504 controls, all of European ancestry. It contains 9,455,779 SNPs. MDD and CHD analysis data were aggregated and available in the IEU Open GWAS database (https://gwas.mrcieu.ac.uk/). See Table 1 for details of the data.

**Table 1:** Details on GWAS of IVs used in Mendelian randomization analyses

Phenotype	ID	Consortium	Sample size	No. of SNPs
MDD	ieu-b-102	PGC	500,199	11,734,353
CHD	ieu-a-7	CARDIoGRAMplusC4D	184,305	9,455,779

*MDD* Major depression disorder, *CHD* Coronary heart disease, *PGC* Psychiatric genomics consortium, *CARDIoGRAMplusC4D* coronary artery disease genome-wide replication and Meta-analysis (CARDIoGRAM) plus the coronary artery disease (C4D) genetics

### Screening SNP

Genetic instruments were selected via the following criteria: (1)* P*-value<$5\times {10}^{-8}$ for association with exposure, (2)a linkage disequilibrium [LD] ${r}^{2}$<0.001, or genetic distance≤10000kb, (3)minimum allele frequency>0.01, (4)removing the SNPs for being palindromic with intermediate allele frequencies, (5)SNP are independent of each other.

After matching the screening and extraction criteria, this study finally identified 47 SNPs associated with MDD and 39 SNPs associated with CHD. SNP number, effective allele, effective allele frequency, effect value, standard error and P value of effective allele with MDD and CHD were extracted as analysis data. See Tables 2 and 3 for details. The β values of the effective alleles with MDD ranged from -0.0620 to 0.0704, and the maximum value of |β| with CHD was 0.0330 (Table 2). For effective alleles with CHD, the range of β values was -0.1809 to 0.3166, and the maximum value of |β| with MDD was 0.0317 (Table 3). The F statistic of all SNPs in this study is greater than 10, indicating that there is no weak instrumental variable bias.

**Table 2:** Detailed information of selected SNPs for MR analysis of the causal effect of MDD on CHD

SNP	Effect allele	Other allele	MDD				CHD			
			eaf	β	se	*P*-value	eaf	β	se	*P*-value
rs1021363	G	A	0.6434	-0.0300	0.0045	2.3E-11	0.6293	-0.0152	0.0098	0.120
rs10235664	C	T	0.2529	-0.0270	0.0049	4.7E-08	0.3020	-0.0208	0.0101	0.039
rs10913112	T	C	0.3780	-0.0262	0.0045	4.5E-09	0.3583	-0.0142	0.0100	0.154
rs12919291	C	G	0.1884	0.0327	0.0055	3.1E-09	0.1711	0.0123	0.0126	0.329
rs12967143	C	G	0.7012	-0.0345	0.0047	2.5E-13	0.6700	-0.0069	0.0100	0.491
rs13037326	T	C	0.2597	0.0310	0.0049	2.4E-10	0.2605	0.0076	0.0104	0.462
rs1367635	C	T	0.5148	0.0253	0.0043	4.4E-09	0.4662	0.0042	0.0095	0.662
rs150186873	C	A	0.0327	0.0704	0.0120	4.5E-09	0.0301	-0.0340	0.0269	0.206
rs150346963	T	C	0.4118	0.0283	0.0044	1.2E-10	0.3990	0.0134	0.0097	0.164
rs17641524	T	C	0.2101	-0.0300	0.0053	1.5E-08	0.1976	0.0199	0.0116	0.086
rs1931388	G	A	0.4042	-0.0295	0.0044	1.7E-11	0.3683	-0.0017	0.0102	0.868
rs1950829	G	A	0.5173	-0.0297	0.0043	4.7E-12	0.4446	-0.0099	0.0096	0.301
rs198457	T	C	0.1886	-0.0315	0.0056	1.9E-08	0.1657	0.0007	0.0138	0.958
rs2111592	A	G	0.3141	0.0263	0.0046	1.3E-08	0.2994	-0.0088	0.0104	0.396
rs2214123	G	A	0.6466	-0.0261	0.0045	8.6E-09	0.6427	-0.0098	0.0100	0.329
rs2232423	G	A	0.1056	-0.0620	0.0070	1.1E-18	0.0864	-0.0204	0.0200	0.308
rs2418449	C	T	0.2810	-0.0281	0.0048	4.2E-09	0.2723	-0.0085	0.0103	0.410
rs247910	G	A	0.4570	0.0237	0.0043	4.7E-08	0.4679	-0.0127	0.0096	0.188
rs2522831	C	T	0.4739	0.0240	0.0043	2.1E-08	0.4828	0.0073	0.0094	0.441
rs2568958	A	G	0.6042	0.0382	0.0044	2.9E-18	0.6149	0.0108	0.0098	0.272
rs28541419	G	C	0.2308	-0.0292	0.0052	1.8E-08	0.2133	-0.0078	0.0123	0.528
rs30266	A	G	0.3271	0.0366	0.0046	1.4E-15	0.3191	0.0070	0.0102	0.491
rs354155	C	G	0.0923	-0.0449	0.0075	1.8E-09	0.1263	0.0044	0.0138	0.747
rs3807865	A	G	0.4105	0.0310	0.0044	1.1E-12	0.4378	0.0275	0.0094	0.004
rs4141983	C	T	0.3260	-0.0264	0.0046	9.7E-09	0.3321	-0.0051	0.0100	0.613
rs4497414	C	T	0.4400	0.0291	0.0044	2.9E-11	0.4326	-0.0102	0.0096	0.286
rs4799949	T	C	0.6684	-0.0292	0.0046	1.4E-10	0.6288	0.0098	0.0096	0.309
rs4936276	C	G	0.6220	0.0278	0.0044	3.6E-10	0.6460	0.0104	0.0101	0.300
rs508502	T	C	0.2992	-0.0264	0.0048	3.6E-08	0.3127	-0.0033	0.0103	0.750
rs59082935	T	C	0.1342	0.0363	0.0066	3.1E-08	0.1708	-0.0071	0.0141	0.614
rs59283172	A	G	0.1081	-0.0390	0.0070	2.4E-08	0.0992	-0.0027	0.0161	0.865
rs61914045	A	G	0.2034	0.0309	0.0054	8.0E-09	0.2104	-0.0045	0.0113	0.688
rs62535714	A	G	0.1639	0.0339	0.0058	4.7E-09	0.1626	0.0091	0.0125	0.465
rs66511648	C	T	0.2840	0.0297	0.0048	6.0E-10	0.2628	-0.0005	0.0108	0.960
rs7152906	C	T	0.5196	0.0258	0.0043	1.9E-09	0.4847	0.0035	0.0093	0.703
rs7241572	A	G	0.2047	0.0323	0.0054	2.4E-09	0.1797	-0.0033	0.0126	0.792
rs72948506	A	G	0.2975	0.0265	0.0047	1.7E-08	0.2649	0.0109	0.0110	0.320
rs7538938	C	T	0.5599	0.0251	0.0043	7.3E-09	0.5775	0.0012	0.0104	0.908
rs754287	A	T	0.3664	-0.0289	0.0045	1.3E-10	0.3896	0.0044	0.0095	0.642
rs7551758	G	T	0.5329	0.0283	0.0043	5.1E-11	0.5244	-0.0068	0.0095	0.472
rs76954012	A	T	0.0931	0.0412	0.0074	2.4E-08	0.0756	0.0303	0.0181	0.094
rs7725715	A	G	0.5343	0.0290	0.0043	1.6E-11	0.5358	-0.0008	0.0093	0.934
rs843812	A	G	0.4117	0.0248	0.0044	1.4E-08	0.4517	-0.0131	0.0096	0.175
rs9364755	G	A	0.2262	0.0283	0.0051	3.5E-08	0.2398	0.0141	0.0107	0.190
rs9529218	T	C	0.2031	-0.0340	0.0054	2.2E-10	0.1951	-0.0101	0.0117	0.388
rs9536381	T	C	0.3259	0.0255	0.0046	2.6E-08	0.2962	0.0050	0.0101	0.621
rs9831648	T	G	0.7739	-0.0292	0.0052	1.6E-08	0.7799	-0.0085	0.0120	0.480

*SNP* Single-nucleotide polymorphism, *MDD* Major depression disorder, *CHD* Coronary heart disease, *eaf* Effective allele frequency, *β* the effect size of exposure or outcome, *se* Standard error

**Table 3:** Detailed information of selected SNPs for MR analysis of the causal effect of CHD on MDD

SNP	Effect allele	Other allele	CHD				MDD			
			eaf	β	se	P-value	eaf	β	se	*P*-value
rs10080815	G	T	0.0276	0.2466	0.0309	1.3E-15	0.0195	0.0114	0.0160	0.477
rs10840293	A	G	0.5498	0.0547	0.0096	1.3E-08	0.5610	0.0028	0.0044	0.529
rs11065979	T	C	0.3655	0.0686	0.0108	1.9E-10	0.4403	-0.0065	0.0043	0.134
rs11191416	G	T	0.1275	-0.0792	0.0135	4.6E-09	0.0882	-0.0317	0.0076	0.000
rs11556924	T	C	0.3133	-0.0726	0.0111	5.3E-11	0.3844	-0.0064	0.0044	0.149
rs115654617	A	C	0.1070	0.1378	0.0158	3.1E-18	0.1264	-0.0118	0.0065	0.067
rs11838776	A	G	0.2633	0.0686	0.0108	1.8E-10	0.2802	-0.0005	0.0048	0.917
rs1199338	C	A	0.1619	0.0736	0.0125	3.9E-09	0.1614	-0.0047	0.0058	0.426
rs12202017	G	A	0.3000	-0.0668	0.0100	2.0E-11	0.2901	0.0058	0.0047	0.222
rs1412444	T	C	0.3691	0.0668	0.0097	5.1E-12	0.3393	-0.0014	0.0045	0.755
rs16986953	A	G	0.1047	0.0852	0.0150	1.5E-08	0.0685	0.0090	0.0086	0.295
rs17087335	T	G	0.2146	0.0608	0.0111	4.6E-08	0.1885	0.0077	0.0055	0.160
rs17678683	G	T	0.0877	0.0988	0.0167	3.0E-09	0.0780	0.0081	0.0081	0.318
rs180803	T	G	0.0293	-0.1809	0.0283	1.6E-10	0.0111	-0.0162	0.0212	0.444
rs1870634	G	T	0.6375	0.0759	0.0097	5.6E-15	0.6656	0.0048	0.0046	0.297
rs2107595	A	G	0.2005	0.0734	0.0113	8.1E-11	0.1560	0.0096	0.0059	0.108
rs2128739	C	A	0.6765	-0.0656	0.0101	7.1E-11	0.7196	-0.0013	0.0048	0.792
rs2487928	A	G	0.4182	0.0626	0.0095	4.4E-11	0.4466	0.0037	0.0043	0.392
rs2519093	T	C	0.1909	0.0797	0.0118	1.2E-11	0.1860	-0.0069	0.0055	0.212
rs2681472	G	A	0.2013	0.0741	0.0113	6.2E-11	0.1708	0.0065	0.0057	0.255
rs28451064	A	G	0.1212	0.1276	0.0160	1.3E-15	0.1308	-0.0017	0.0066	0.800
rs2891168	G	A	0.4887	0.1934	0.0092	2.3E-98	0.4883	0.0085	0.0043	0.047
rs3918226	T	C	0.0645	0.1333	0.0221	1.7E-09	0.0803	-0.0004	0.0082	0.963
rs4420638	G	A	0.1660	0.0919	0.0141	7.1E-11	0.1865	-0.0009	0.0056	0.872
rs4468572	C	T	0.5858	0.0772	0.0095	4.4E-16	0.5736	0.0055	0.0044	0.208
rs4593108	G	C	0.2047	-0.0708	0.0116	8.8E-10	0.1726	-0.0022	0.0057	0.703
rs515135	C	T	0.7920	0.0675	0.0122	3.1E-08	0.8206	-0.0008	0.0056	0.885
rs55730499	T	C	0.0562	0.3166	0.0242	5.4E-39	0.0778	-0.0024	0.0081	0.770
rs56062135	T	C	0.2057	-0.0697	0.0119	4.5E-09	0.2362	-0.0047	0.0051	0.356
rs56289821	A	G	0.1004	-0.1336	0.0170	4.4E-15	0.1169	0.0040	0.0067	0.548
rs56336142	C	T	0.1927	-0.0668	0.0119	1.8E-08	0.2124	0.0124	0.0053	0.019
rs663129	A	G	0.2568	0.0582	0.0105	3.2E-08	0.2352	-0.0159	0.0051	0.002
rs6689306	G	A	0.5525	-0.0560	0.0094	2.6E-09	0.5810	-0.0021	0.0044	0.642
rs67180937	G	T	0.6631	0.0788	0.0111	1.0E-12	0.7354	-0.0011	0.0050	0.820
rs7212798	C	T	0.1465	0.0800	0.0142	1.9E-08	0.1485	-0.0003	0.0061	0.960
rs7528419	G	A	0.2142	-0.1145	0.0115	2.0E-23	0.2217	-0.0004	0.0052	0.931
rs8042271	A	G	0.0977	-0.0967	0.0176	3.7E-08	0.0385	-0.0012	0.0113	0.916
rs9349379	G	A	0.4316	0.1318	0.0097	1.8E-42	0.4066	-0.0025	0.0044	0.562
rs9970807	T	C	0.0849	-0.1258	0.0167	5.0E-14	0.0909	0.0019	0.0075	0.799

*SNP* Single-nucleotide polymorphism, *MDD* Major depression disorder, *CHD* Coronary heart disease, *eaf* Effective allele frequency, *β* the effect size of exposure or outcome, *se* Standard error

### Statistical analysis

MR analysis must be preceded by statistical tests for heterogeneity among SNPs in order to avoid biased estimates of causal effects. The ${I}^{2}$ statistics reflected SNPs heterogeneity's contribution to the overall variance of the effect size, and ${I}^{2}$=$\frac{(Q-df)}{Q}\times 100\mathrm{\%}$. Q was the normalized sum of squares of effect size, and $df$ was the degree of freedom. ${I}^{2}$ for 0%-25%, 25%-50%, and>50% represented the presence of mild, severe, and high heterogeneity, respectively.

We considered four main methods for calculating two-sample Mendelian randomized causal associations. (1) Wald ratios method: It was mainly used to calculate causal effect values when a single SNP as IV, i.e. ${\beta }_{\mathrm{IV}}={\beta }_{\mathrm{GY}}\div {\beta }_{\mathrm{GX}}$, ${\beta }_{\mathrm{GY}} \mathrm{and} {\beta }_{\mathrm{GX}}$ are the effective values of genetic variation and outcome, and genetic variation and exposure, respectively. (2) Inverse-variance weighting (IVW): It was based on forcing the intercept term in weighted linear regression to zero in order to determine the causal effect value, and all three assumptions must be met for the causal effect estimate to be unbiased. IVW could be used to integrate multiple SNPs to get a consistent causal effect value estimate [12]. (3) MR-Egger regression: In contrast to IVW forced linear regression, MR-Egger used intercept terms to measure the multiplicity of effects among instrumental variables. This intercept term could be interpreted as an estimate of genetic variances multiplied by the mean variation. Therefore, MR-Egger regression could be used to determine whether horizontal pleiotropy was present or absent. In the meantime, MR-Egger weakly assumed that the MR-Egger regression model coefficients could be used to estimate an unbiased causal effect of exposure on the outcome by satisfying merely the assumption that IV's direct effect on outcome was independent of IV's association with exposure (instrument strength independent of direct effect, InSIDE) [13]. (4) Weighted median method: With at least 50% of the SNPs valid as instrumental variables, the weighted median method produced strong causal effect values [14].

Sensitivity analysis was an essential link in MR Analysis. We used the leave-one-out method to remove an SNP one by one and re-integrated IV with the remaining SNPs to calculate the OR value of causal effect, based on which the effect size of the removed SNPs on the results was evaluated.

All analyses were performed using R (version 4.2.1; https://www.r-project.org/) and R studio (version 2022.071–554; https://rstudio.com/products/rstudio/) to complete. Statistical significance was defined as *P* < 0.05.

## Results

### The casual effect of MDD on CHD

**Fig. 2:**
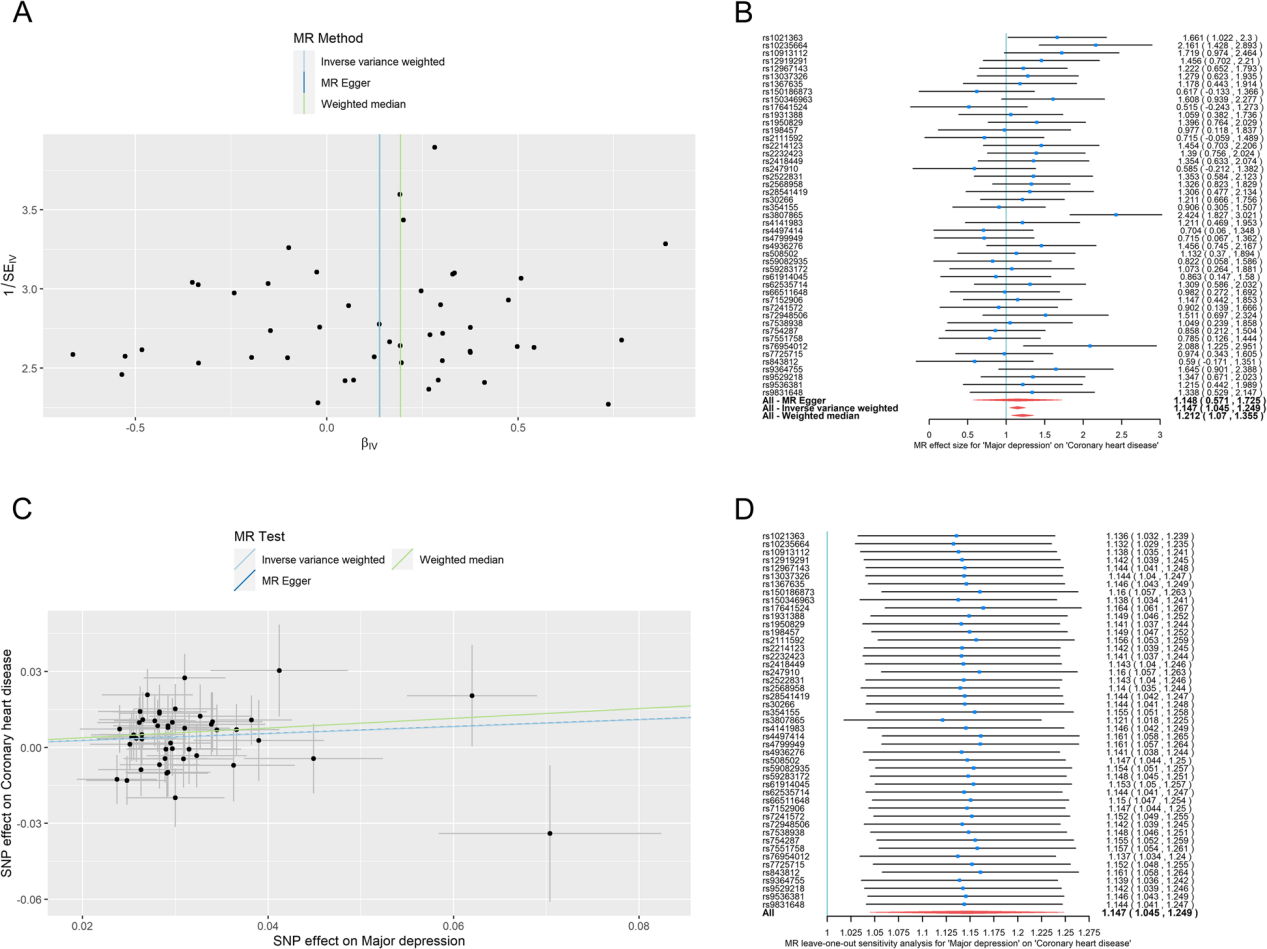
The forward MR analyses: Casual effect of MDD on CHD. **A** A funnel plot was applied to detect whether the observed association was along with obvious heterogeneity. **B** A forest plot was used to show the OR value and 95% CI value (black line segments) for each SNP and show the MR‐Egger, IVW, and weighted median results at the bottom. **C** Scatter plot of the association between MDD and CHD. The three methods applied in the current manuscript were all depicted. Lines in light blue, dark blue, and green represent IVW, MR‐Egger, and weighted median. **D** Leave‐one‐out analyses to evaluate whether any single instrumental variable was driving the causal effect. MDD, major depression disorder; CHD, coronary heart disease; IVW, inverse variance weighted; MR, mendelian randomization

The heterogeneity test results of IVW and MR-Egger (Table 4, all *P*-values of Cochran's Q > 0.05, ${I}^{2}$=0), as well as the funnel plot (Fig. 2A), showed that there was no heterogeneity among SNPs. When SNP was used as IV, the OR value of MDD for CHD was between 0.515 and 2.424 (Fig. 2B). SNPs rs1021363, rs10235664, rs3807865, and rs76954012 as instrumental variables were statistically significant (*P* < 0.05), whereas no significant difference was observed between the effect values of the other SNPs (*P* > 0.05). When integrating 47 SNPs into IV, the MR-Egger regression model's intercept term was -0.0000376, *P* = 0.996 > 0.05, and there was no horizontal pleiotropy (Table 4). The results of IVW provided consistent estimates of the causative influence of MDD and CHD. The IVW results demonstrated an OR of 1.147 (95% CI: 1.045, 1.249) for MDD on CHD, confirming a causal effect of MDD on CHD (Table 5), which suggested that having MDD increases the probability of acquiring CHD by 14.7%. The weighted median method produced a similar result (OR = 1.212, 95% CI: 1.054 to 1.394), indicating a positive causal relationship between MDD and CHD (Table 5). The cohort did not satisfy the InSIDE assumption for MR-Egger regression. Hence the MR-Egger results were skewed. The scatter plot of the effect values of each SNP on MDD and CHD also revealed a positive causal relationship between MDD and CHD (Fig. 2C). Furthermore, after leaving out rs1021363 from 47 SNPs in a Leave-one-out analysis, the remaining 46 SNPs were reintegrated as IVs, and IVW determined the OR of MDD on CHD to be 1.136 (95% CI: 1.032 1.239, P0.05), indicating that this SNP did not contribute significantly to the estimation of causal effects. Despite removing other SNPs, the results remained robust, and no SNP significantly impacted causal effect estimation (Fig. 2D).

**Table 4:** Heterogeneity test and horizontal pleiotropy test

Exposure/Outcome	Heterogeneity test (IVW)			Heterogeneity test (MR-Egger)			Horizontal pleiotropy test (MR-Egger)		
	Q	$\boldsymbol I^{\mathbf2}$	*P*-value	Q	$\boldsymbol I^{\mathbf2}$	*P*-value	Intercept	se	*P*-value
MDD/CHD	42.28	0	0.629	42.28	0	0.588	-3.76E-5	0.0089	0.997
CHD/MDD	62.48	39.18%	0.007	62.46	40.76%	0.006	-0.0003	0.0027	0.920

*MDD* Major depression disorder, *CHD* Coronary heart disease, *IVW* Inverse variance weighted, *se* Standard error

**Table 5:** Bidirectional MR results between MDD and CHD

Methods	Influence of MDD traits on CHD		Influence of CHD traits on MDD	
	OR (95%CI)	β	OR (95%CI)	β
IVW	1.147(1.045,1.249)	0.137	1.008(0.985,1.031)	0.008
IVW(re)			1.008(0.985,1.031)	0.008
MR-Egger	1.148(0.571,1.725)	0.138	1.011(0.957,1.064)	0.011
Weighted median	1.212(1.070,1.355)	0.192	0.994(0.967,1.021)	-0.006

*MDD* Major depression disorder, *CHD* Coronary heart disease, *IVW* Inverse variance weighted, *IVW(re)* Inverse variance weighted of random effects

### The casual effect of CHD on MDD

**Fig. 3:**
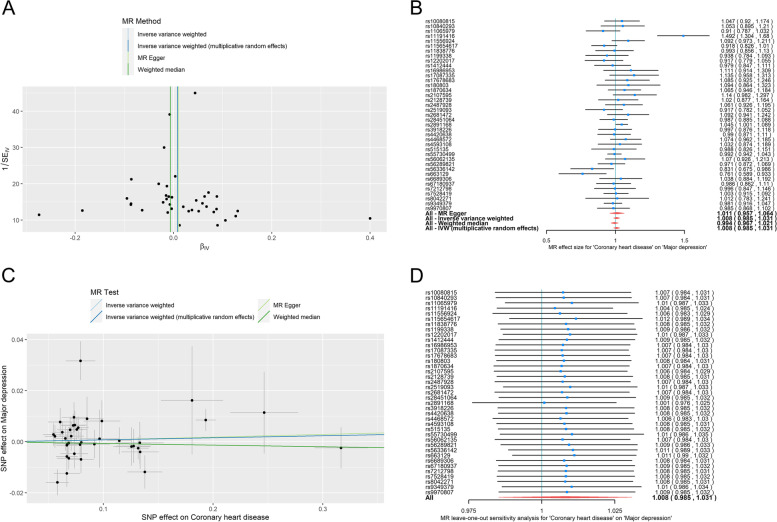
The reverse MR analyses: Casual effect of CHD on MDD. **A** A funnel plot was applied to detect whether the observed association was along with obvious heterogeneity. **B** A forest plot was used to show the OR value and 95% CI value (black line segments) for each SNP and show the MR‐Egger, IVW, weighted median, and IVW (multiplicative random effects) results at the bottom. **C** Scatter plot of the association between CHD and MDD. The three methods applied in the current manuscript were all depicted. Lines in light blue, dark blue, light green, dark green represent IVW, IVW (multiplicative random effects), MR‐Egger, and weighted median. **D** Leave‐one‐out analyses to evaluate whether any single instrumental variable was driving the causal effect. MDD, major depression disorder; CHD, coronary heart disease; IVW, inverse variance weighted; MR, mendelian randomization

The heterogeneity test results for IVW and MR-Egger (Table 4, all P-values of Cochran's Q 0.05, I2 = 39.18%) suggested that SNPs were heterogeneous. Similarly, it was possible to observe that the rightmost point deviates significantly from the mean, indicating the existence of heterogeneity among SNPs (Fig. 3A). We used a random-effects model to estimate the causal effect estimates for CHD and MDD, taking into account the substantial heterogeneity of these 39 SNPs. The OR of CHD to MDD ranged between 0.760 and 1.492 when using SNP as IV (Fig. 3B). SNPs rs11191416, rs56336142, and rs663129 as instrumental variables were statistically significant (*P* < 0.05), whereas no significant difference was observed between the effect values of the other SNPs (*P* > 0.05). The intercept term of the MR-Egger regression model when integrating 39 SNPs into IV was -0.0002698, *p* = 0.920 > 0.05, and horizontal pleiotropy was absent (Table 4). Estimates of the causal effect of CHD and MDD were consistently based on the results of IVW utilizing the random effects model. The OR of 1.008 (95% CI: 0.985, 1.031) for CHD against MDD did not demonstrate a causal relationship between CHD and MDD (Table 5). The results of the weighted median method similarly corroborated this conclusion (OR = 0.99, 95% CI: 0.966–0.100), demonstrating no causal association between CHD and MDD (Table 5). While the cohort did not support the InSIDE hypothesis for ME-Egger regression, the MR-Egger results were biased. The scatter plots of the effect values of each SNP on CHD and MDD revealed a substantial difference between the causal effect values estimated by different MR analysis methods. Therefore, it was possible to infer that there was no causal association between CHD and MDD (Fig. 3C). In order to verify the robustness of the model, we performed the Leave-one-out method to exclude rs10080815 from 39 SNPs and reintegrated the remaining 38 SNPs as IV. According to the random effects model of IVW, the OR of CHD on MDD was 1.007 (95% CI: 0.984 ~ 1.031, *P* > 0.05), indicating that changing this SNP would not significantly affect the estimation of a causal effect. The results were likewise robust after excluding other SNPs, and no SNPs had a substantial impact on the causal effect estimations (Fig. 3D).

## Discussion

The study presents the bidirectional MR to research the genetic association of MDD and CHD. We observed that there was indeed a one-way causal association between MDD and CHD, where MDD was a risk factor for developing CHD, while CHD had no statistically significant effect on MDD.

The results of the present study indicated that individuals with MDD had a 14.7% increased risk of developing CHD (OR = 1.147, 95% CI:1.045–1.249), which was in line with some epidemiological findings. Epidemiological research has revealed that MDD was an independent (i.e., confounder-adjusted) risk factor for morbidity and mortality in CHD. A meta-analysis of 30 prospective cohort studies (*N* = 893,850) discovered that people with MDD had a 30% greater risk of getting CHD than patients without MDD (RR = 1.30, 95% CI:1.22–1.34) [15]. Similarly, another meta-analysis of prospective cohort studies (*N* = 323,709) found that MDD was associated with an increased risk of coronary death by 36% compared with non-MDD patients (adj-HR = 1.36, 95% CI:1.14–0.63) [16]. Additionally, a number of epidemiological studies have identified CHD as a risk factor for MDD. One study indicated that the prevalence of MDD in patients with CHD was much greater than in the general population, with more than one-fifth of individuals with CHD having MDD and up to one-third reporting heightened symptoms of MDD [17]. However, no causative association between CHD and MDD was identified in our study, and which was no direct evidence that CHD increases the chance of developing MDD. Therefore, we hypothesized that unidentified confounding factors might have influenced the conclusions of the epidemiological study.

It was unclear how MDD and CHD are causally related, and the association might involve both causality and common physiological pathways [18]. The underlying mechanisms by which MDD causes CHD were complex and not fully understood. Several categories of putative routes for elevated CHD risk in MDD patients have been identified by studies: Biological, psychological, behavioral, and genetic mechanisms [19]. Inflammation plays an essential role in the pathogenesis of MDD and CHD. Complete blood counts (CBCs) were simple and sensitive indicators of inflammatory changes in the body, including white blood cells (WBC), monocytes, neutrophils, lymphocytes, neutrophil/lymphocyte ratios (NLR), platelet/lymphocyte ratios (PLR), monocyte/lymphocyte ratios (MLR), and systemic immune-inflammatory indexes (SIII) so on. Shafiee et al. found that higher depression scores were associated with an enhanced inflammatory state characterized by elevated WBC [20]. Euteneuer et al. further discovered that MDD patients had higher monocytes and NLR levels than controls [21]. There were indications that CBC, a marker of inflammation, might have a role in the pathophysiology of MDD [22]. Furthermore, it was revealed in another study that these inflammatory markers had a positive correlation with cardiac conditions such as congestive heart failure, coronary artery disease, and myocardial infarction [23]. As a result, the elevated levels of inflammatory markers in patients might be affected by MDD, which increases the risk of coronary heart disease. As for daily life habits, patients with MDD tended to be poor health behaviors, including smoking, low physical activity and eating poorly [24]. These unhealthy behaviors increased their chances of developing obesity, diabetes, and heart attacks, all of which were risk factors for CHD [25]. Moreover, people suffering from MDD were likely to be isolated from others and had difficulties receiving good social support. Social isolation has been shown to contribute to cardiac disease significantly and even increase mortality rates. Objectively, a 2-to threefold rise in the frequency of coronary heart disease over time was linked to weaker social support networks. Patients reported that a lack of emotional support also raised the likelihood of subsequent unfavorable cardiac events. Consequently, the psychological effect of MDD leads to an increased risk of CHD for patients [26]. According to genetic research, MDD and cardiometabolic illness were strongly inherited [27]. Despite genetic factors contributing 30%—60% to MDD and 30%—60% to CHD, T win study and the molecular genetic study revealed a relatively modest genetic correlation between cardiometabolic abnormalities, coronary heart disease, and MDD. It seemed that pleiotropy was associated with shared genetic loci in MDD and cardiometabolic diseases [28].

This study is significantly different from previous similar studies. Rukh G et al. ' s study used neuroticism scores to divide the study population into three genetic subgroups (depression, worry, and sensitivity to environmental stress and adversity [SESA]), and explored their potential two-way causal relationship with multiple cardiovascular diseases. It is a multi-exposure to multi-outcome MR study. The study found that people with higher neuroticism scores were more likely to have depressive symptoms, and the risk of heart failure 1.32 (1.12–1.56) and myocardial infarction 1.47 (1.18–1.83) was also higher [29]. However, high neuroticism scores are not the clinical diagnostic criteria for depression, so the results are biased. The study of Lu Y et al. explored the causal relationship between genetic susceptibility to depression and a variety of cardiovascular diseases. It is a MR study of single exposure to multiple outcomes. This study supports the causal relationship between the genetic risk of depression and the risk of CAD, myocardial infarction, heart failure and small vessel stroke, and suggests that some of the causal relationship is mediated by type 2 diabetes and smoking. In the study of Lu Y, a variety of cardiovascular diseases are regarded as the outcome, which has a certain degree of sample overlap, resulting in the causal estimation results between MDD and CHD may be affected by other cardiovascular diseases and deviate from the real situation. Our study directly focused on MDD and CHD, and performed reverse MR analysis, making our results more credible, which is significantly different from Lu Y 's study. On the other hand, Lu Y selected three GWAS studies with different definitions of depression, which weakened the causal effect of genetic tools [30]. The GWAS data selected in this study have a clear definition of MDD and CHD, and there is no risk of weakening the causal effect.

There were several advantages to this study. Firstly, a bidirectional MR study demonstrated a stronger correlation between MDD and CHD. Furthermore, the MR method provided accurate estimates of causal effects while considering confounding factors and reverse causality. Finally, the F-statistics of the instrumental variables included in this study were all greater than 10, no weak instrumental variable bias was detected, and sensitivity analysis was used to ensure that the causal effect results obtained were sufficiently robust. Nevertheless, this study was limited to the European ethnicity, which lacked diversity and made it difficult to generalize to other ethnicities, so more studies should be conducted. In addition, no corresponding population follow-up data available to corroborate the findings on an epidemiological level.

## Conclusion

In this study, we suggested a unidirectional causal association between MDD and CHD, with MDD causing increase in the risk of CHD. But in turn, there is insufficient evidence that CHD causes MDD. Therefore, the influence of psychological factors should also be considered in the prevention and treatment of CHD. For MDD patients, it is necessary to prevent cardiovascular diseases.

## Data Availability

Publicly available datasets were analyzed in this study. All datasets for this study are included in the article. The authors acknowledge the efforts of all of the researchers who have contributed the data to the public databases of IEU Open GWAS (https://gwas.mrcieu.ac.uk/).
